# Multiple fibroepitheliomas of Pinkus after radiotherapy^☆^^☆☆^

**DOI:** 10.1016/j.abd.2019.09.020

**Published:** 2019-09-30

**Authors:** Bruna Anjos Badaró, Lucia Martins Diniz, Ernesto Negris Neto, Elton Almeida Lucas

**Affiliations:** a Service of Dermatology, Hospital Universitário Cassiano Antônio Moraes, Universidade Federal do Espírito Santo, Vitória, ES, Brazil; b Department of Clinical Medicine – Dermatology, Universidade Federal do Espírito Santo, Vitória, ES, Brazil; c Service of Dermatopathology, Hospital Universitário Cassiano Antônio Moraes, Universidade Federal do Espírito Santo, Vitória, ES, Brazil

Dear Editor,

Fibroepithelioma of Pinkus (FEP), also known as fibroepithelial basal cell carcinoma, is a rare cutaneous neoplasm. It has a frequency that ranges from 0.2% to 1.4% in a series of basal cell carcinomas.^1^, ^2^ Its etiopathogenesis is controversial; some authors consider it a variant of basal cell carcinoma, and others, as a variant of trichoblastoma.^2^ However, there are studies that suggest a significant influence of previous exposure to radiotherapy.^1^, ^3^ Based on clinical suspicion, the peculiar and unmistakable histopathology confirms the diagnosis.^2^ The present study reports the case of man exhibiting neoplastic cutaneous lesions, restricted to the site in the left thigh where radiotherapy had been previously administered for bone tumor.

The patient was 69 years old, male, white, and with a history of bone tumor in the left femur at the age of 17 years. He had undergone a surgical intervention and adjuvant radiotherapy. Years later, he noticed the appearance of red-brown, asymptomatic, slowly evolving papules, restricted to the skin adjacent to the surgical scar, in the area previously subjected to radiotherapy. In 2016, the patient sought dermatological treatment. He exhibited discreetly raised translucent erythematous plaques with pigmented inferior borders, located on the upper lateral aspect of the left thigh. He also exhibited some translucent erythematous papules, located near the surgical scar (Fig. 1). The histopathological study of the excised plaque from the upper left thigh exhibited neoplastic basaloid cells infiltrating the dermis, with anastomoses that had a cord-like appearance, accompanied by surrounding desmoplasia, and compatible with FEP (Fig. 2). All other lesions in the irradiated area were excised and submitted to histopathological examination, maintaining the diagnosis of FEP characterized by multiple lesions.

**Figure 1:**
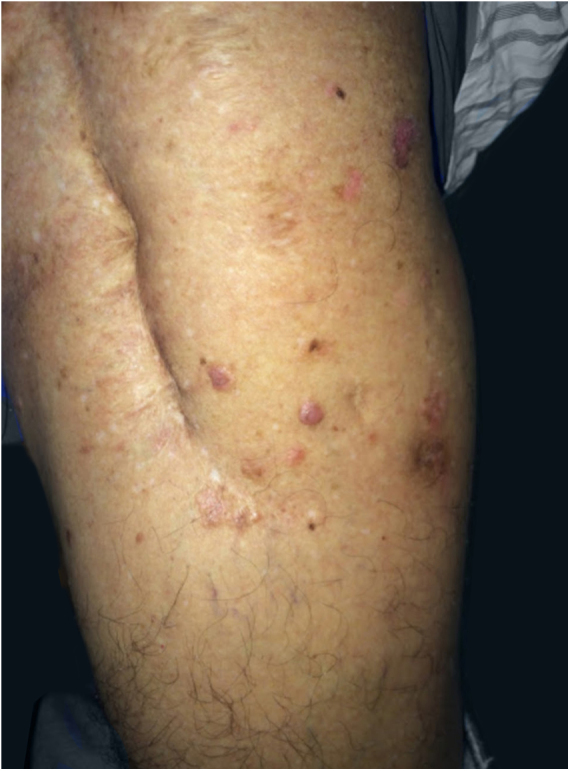
Presence of translucent erythematous papules, discreetly raised red-brown plaques adjacent to the surgical scar in the left thigh. Anterolateral view.

**Figure 2:**
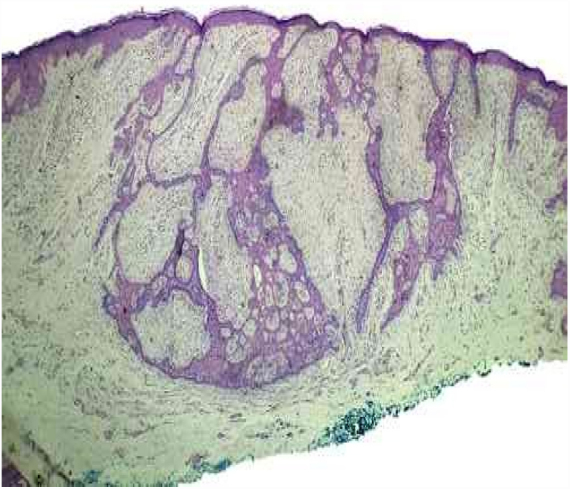
Epithelial proliferation infiltrating the dermis, with anastomoses that had a cord-like appearance, accompanied by surrounding desmoplasia (hematoxylin & eosin, ×4).

FEP affects non-photoexposed areas, commonly in the inguinal and lumbosacral regions.^1^, ^2^ It occurs in individuals aged between 40 and 60 years,^1^ with a discrete predominance in women (54%).^2^ It is clinically characterized by single or multiple lesions, such as papules or plaques, which are normochromic/brownish, cupuliform, or sessile.^1^, ^2^ The case reported diverges with respect to sex (male); however, it is similar regarding location (non-photoexposed area), age (69 years), and clinical manifestation (multiple discreetly raised erythematous plaques and red-brown papules).

The etiopathogenesis of FEP is controversial.^2^ Some authors classify it as a variant of basal cell carcinoma, and others as a variant of trichoblastoma.^1^ Evidence in the literature remains difficult to classify, in some aspects favoring basal cell carcinoma, and, in others, trichoblastoma.^2^ However, it has been argued that there is an association between FEP and previous exposure to radiotherapy,^1^, ^3^ as in the case reported, in which the patient's neoplastic lesions were restricted to the previously irradiated region. In a literature review, four articles were found referring to the association between FEP and previously irradiated skin areas.^1^, ^3^, ^4^, ^5^

The histopathological features are fundamental for diagnosis. They are described as peculiar and unmistakable, evidencing thin anastomosing strands of basaloid cells, surrounded by abundant stroma, which form a uniform border with the underlying dermis, resembling a honeycomb.^1^, ^2^ These characteristics were found in the present case.

The evolution of FEP is slow. It exhibits low local aggression and little risk of metastasis.^1^, ^2^ Excisions of the lesions are recommended and these procedures are almost always curative, given that topical treatments are ineffective.^1^ Patients should attend dermatological follow-up appointments for early diagnosis and treatment of new lesions.

In the presented case, the patient had multiple FEP lesions excised and is attending regular follow-up appointments at the dermatology service.

This case is reported to emphasize the possibility of other etiologies of basal cell carcinomas, such as previous radiotherapy.

## Financial support

None declared.

## Author's contributions

Bruna Anjos Badaró: Conception and planning of the study; elaboration and writing of the manuscript; obtaining, analyzing and interpreting the data; critical review of the literature; critical review of the manuscript.

Lucia Martins Diniz: Approval of the final version of the manuscript; conception and planning of the study; effective participation in research orientation; intellectual participation in propaedeutic and/or therapeutic conduct of the cases studied; critical review of the literature; critical review of the manuscript.

Ernesto Negris Neto: Obtaining, analyzing and interpreting the data; intellectual participation in propaedeutic and/or therapeutic conduct of the cases studied.

Elton Almeida Lucas: Obtaining, analyzing and interpreting the data; effective participation in research orientation; intellectual participation in propaedeutic and/or therapeutic conduct of the cases studied.

## Conflicts of interest

None declared.
